# Prevention of Apoptosis by Mitochondrial Phosphatase PGAM5 in the Mushroom Body Is Crucial for Heat Shock Resistance in *Drosophila melanogaster*

**DOI:** 10.1371/journal.pone.0030265

**Published:** 2012-02-07

**Authors:** Yosuke Ishida, Yusuke Sekine, Haruka Oguchi, Takahiro Chihara, Masayuki Miura, Hidenori Ichijo, Kohsuke Takeda

**Affiliations:** 1 Laboratory of Cell Signaling, Graduate School of Pharmaceutical Sciences, The University of Tokyo, Tokyo, Japan; 2 Department of Genetics, Graduate School of Pharmaceutical Sciences, The University of Tokyo, Tokyo, Japan; 3 Precursory Research for Embryonic Science and Technology (PRESTO), Japan Science and Technology Agency (JST), Saitama, Japan; Oregon Health and Science University, United States of America

## Abstract

The heat shock (HS) response is essential for survival of all organisms. Although the machinery of the HS response has been extensively investigated at the cellular level, it is poorly understood at the level of the organism. Here, we show the crucial role of the mushroom body (MB) in the HS response in *Drosophila*. Null mutants of the mitochondrial phosphatase *Drosophila* PGAM5 (dPGAM5) exhibited increased vulnerability to HS, which was reversed by MB-specific expression of the caspase inhibitor p35, and similar vulnerability was induced in wild-type flies by knockdown of MB *dPGAM5*. Elimination of the MB did not affect the HS response of wild-type flies, but did increase the resistance of dPGAM5-deficient flies to HS. Thus, the MB may possess an apoptosis-dependent toxic function, the suppression of which by dPGAM5 appears to be crucial for HS resistance.

## Introduction

Increases in temperature threaten all living organisms due to heat shock (HS) stress. In order to adapt to or resist HS stress, the cellular expression of a series of HS proteins (HSPs) is induced. Most of HSPs act as molecular chaperones that prevent protein aggregation and support proper protein folding [1]. However, cells undergo apoptosis or necrosis when subjected to severe and/or prolonged HS stress that exceeds the capacity of the protective HS response via HSPs [2]. The mechanisms of HS-induced apoptosis have been the focus of intense investigation and are generally well characterized in cultured mammalian cells. For instance, among caspases, caspase-2 and caspase-9 have been proposed to act as initiator caspases and to mediate HS-induced apoptosis via mitochondrial outer-membrane permeabilization [3], [4]. However, at the level of the whole organism, the significance of cellular apoptosis in the HS response has not been as intensively investigated. Model animals such as *Caenorhabditis elegans* and *Drosophila melanogaster* are expected to provide useful whole-body assay systems. Indeed, HS-induced apoptosis has already been clearly demonstrated in the *C. elegans* germline and the *Drosophila* wing imaginal disc [5], [6].

Phosphoglycerate mutase 5 (PGAM5) is a member of the PGAM family and has been reported to be localized to the mitochondria through its N-terminal transmembrane domain [7]. PGAM5 has been shown to interact with the anti-apoptotic protein Bcl-xL and the BTB-Kelch substrate adaptor protein Keap1, which is functional in the Cul3-dependent ubiquitin ligase complex [8], [9]. It was recently demonstrated that PGAM5 lacks authentic PGAM activity but possesses protein phosphatase activity highly specific to serine and threonine residues. Furthermore, it has also been found that, depending on its phosphatase activity, PGAM5 activates ASK1, the upstream regulator of the stress-activated c-Jun N-terminal kinase (JNK) and p38 MAP kinase pathways [10]. More recently, the *Drosophila* ortholog of mammalian PGAM5 (dPGAM5) has been shown to act as an exacerbating factor in the *Drosophila* model of Parkinson's disease induced by mutation of PTEN-induced kinase 1 (PINK1), a mitochondrial serine/threonine kinase known as a gene product responsible for early-onset autosomal recessive Parkinson's disease [11]. In this report, we analyzed the response of dPGAM5-deficient flies to HS stress and found that the mushroom body (MB) in the brain and dPGAM5 therein play a crucial role in the whole-body response to HS.

## Results

### Flies deficient in dPGAM5 are vulnerable to HS

We previously generated a *dPGAM5* null allele, *PGAM5^1^*, and found that *PGAM5^1^* hemizygous (*PGAM5^1^/Y*) flies, designated here as *PGAM5^1^* flies, were viable and fertile but showed a slightly longer lifespan than control (*y^1^w^1^/Y*) flies [11]. Recently, we observed that *PGAM5^1^* flies showed a similar lifespan to that of control flies when maintained on a glucose-based diet instead of the molasses-based diet that had previously been used (Fig. S1). This condition was favorable for phenotypic analyses of *PGAM5^1^* flies, since differences in lifespan may bias the evaluation of the responses of *PGAM5^1^* flies to environmental stressors.

**Figure 1:**
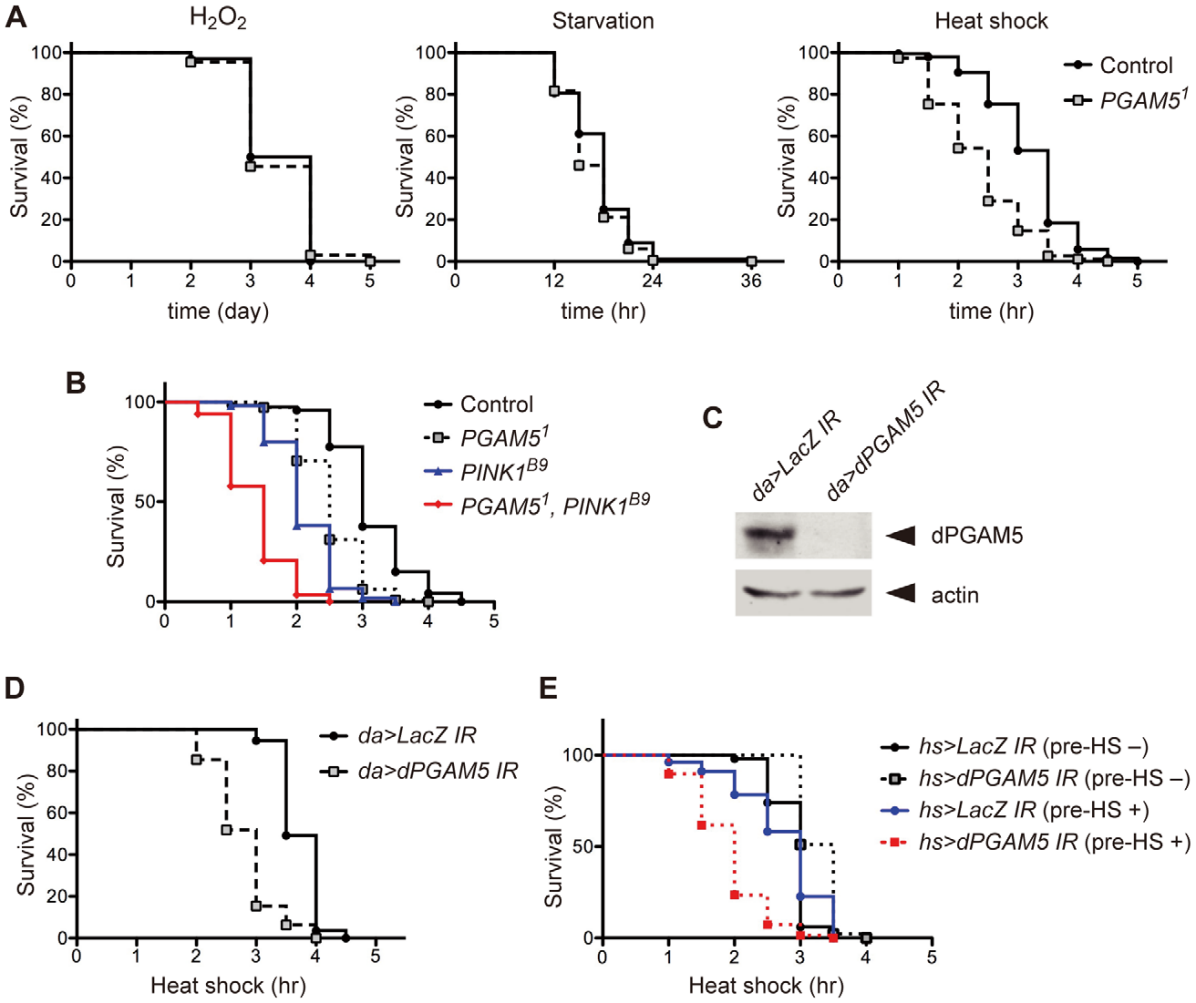
Flies deficient in dPGAM5 are vulnerable to HS. (**A**) *PGAM5^1^* flies are vulnerable to HS. Survival curves of adult male control (*y^1^w^1^/Y*) and *PGAM5^1^* flies (*y^1^*, *PGAM5^1^/Y*) subjected to oxidative stress (0.1% H_2_O_2_, *n* = 66; left), starvation (*n* = 180; middle), or HS (37°C, *n* = 190; right) are shown. Control vs. *PGAM5^1^* in HS, *p*<0.0001 by the log-rank test. (**B**) dPGAM5 and dPINK1 act independently in HS response. Survival curves of the indicated adult male flies (day 5–15) subjected to HS are shown (control, *n* = 120; *PGAM5^1^*, *n* = 112; *PINK1^B9^*, *n* = 105; *PINK1^B9^, PGAM5^1^*, *n* = 116). *PINK1^B9^* vs *PGAM5^1^, PINK1^B9^*, p<0.0001 by the log-rank test. The genotypes are revertant *PINK1^REV^/Y* (Control), *PGAM5^1^/Y* (*PGAM5^1^*), *PINK1^B9^/Y* (*PINK1^B9^*) and *PGAM5^1^, PINK1^B9^/Y* (*PGAM5^1^, PINK1^B9^*). (**C**) Efficiency of IR-mediated *dPGAM5* knockdown. Protein expression of dPGAM5 in *daughterless (da)>LacZ IR* flies (*da-GAL4/+; UAS-LacZ IR/+*) and *da>dPGAM5 IR* flies (*da-GAL4/+*; *UAS-dPGAM5 IR/+*) was detected by immunoblotting with dPGAM5 antibody. Actin was also detected as a loading control. (**D**) Knockdown of *dPGAM5* in the whole body induces vulnerability of flies to HS. Survival curves of adult male *da>LacZ IR* flies and *da>dPGAM5 IR* flies subjected to HS are shown (*n* = 110). *p*<0.0001 by the log-rank test. (**E**) Transient knockdown of *dPGAM5* prior to HS is sufficient to induce vulnerability of flies to HS. Survival curves of the indicated adult male flies subjected to HS are shown. IR RNA was induced two days prior to sustained HS by two cycles of HS pretreatment, each of which was composed of 37°C for 30 min, 25°C for 5 hr, 37°C for 30 min and 25°C for 18 hr. “Pre-HS +” and “pre-HS −” indicate flies expressing and not expressing IR RNA, respectively, when they were subjected to sustained HS. The genotypes are *hs-GAL4/UAS-LacZ-IR* (*hs>LacZ-IR*) and *hs-GAL4/UAS-dPGAM5-IR* (*hs>dPGAM5-IR*). *hs>LacZ-IR* (pre-HS +) flies (*n* = 79), *hs>dPGAM5-IR* (pre-HS +) flies (*n* = 68), *hs>LacZ-IR* (pre-HS −) flies (*n* = 50) and *hs>dPGAM5-IR* (pre-HS −) flies (*n* = 45) were examined. *hs>LacZ-IR* (pre-HS +) vs *hs>dPGAM5-IR* (pre-HS +), p<0.0001 by the log-rank test.

Although *PGAM5^1^* flies showed neither resistance nor sensitivity to oxidative stress (H_2_O_2_) and starvation, they were sensitive to HS stress and died sooner than control flies (Fig. 1A), suggesting that dPGAM5 is required for proper response to HS. Since it was previously found that introduction of the *PGAM5^1^* allele into a loss-of-function allele for *Drosophila PINK1* (*dPINK1*), *PINK1^B9^*, ameliorated pathological changes induced by dPINK1 deficiency [11], we examined the genetic interaction between *dPGAM5* and *dPINK1* in the context of HS response. As shown in Fig. 1B, *PINK1^B9^* flies were also sensitive to HS stress and died even sooner than *PGAM5^1^* flies. Importantly, however, *PGAM5^1^, PINK1^B9^* double mutant flies were more sensitive to HS stress than either *PGAM5^1^* flies or *PINK1^B9^* flies, suggesting that dPGAM5 and dPINK1 are involved in HS response but act independently of each other.

To confirm and further analyze the involvement of dPGAM5 in HS response, we used transgenic flies harboring the gene encoding an inverted repeat (IR) RNA for *dPGAM5*, which specifically inhibits *dPGAM5* expression, downstream of the UAS binding sequence for the transcription factor GAL4 (*UAS-dPGAM5 IR*). When these flies were crossed with *daughterless (da)-GAL4*, a driver strain that expresses GAL4 ubiquitously, the progeny (*da>dPGAM5 IR*) in which *dPGAM5* was knocked down in the whole body was also vulnerable to HS (Fig. 1C, D). To exclude the possibility that persistent deficiency of dPGAM5 in flies throughout development affects their HS sensitivity, we transiently knocked down *dPGAM5* prior to the HS treatment using *heat shock (hs)>dPGAM5 IR* flies in which *dPGAM5* was knocked down by four times transient treatment of flies at 37°C each for 30 min. These flies were vulnerable to sustained HS, whereas the sensitivity of the flies without HS pretreatment, thus not expressing *dPGAM5 IR*, was similar to that of control *hs>LacZ IR* flies (Fig. 1E). This result suggests that dPGAM5 is directly involved in HS response in adult flies.

### A deficiency of dPGAM5 in the MB induces vulnerability to HS

**Figure 2:**
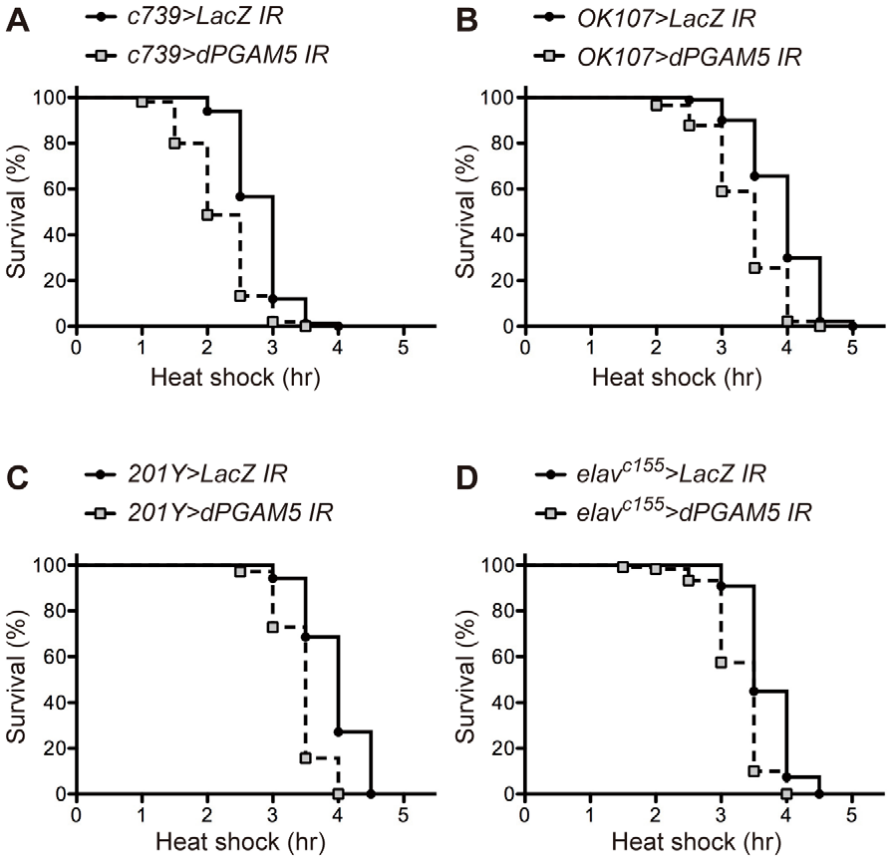
Deficiency of dPGAM5 in the MB induces vulnerability to HS. Knockdown of *dPGAM5* in the MB (**A–C**) and pan-neuronal knockdown of *dPGAM5* (**D**) induce vulnerability of flies to HS. Survival curves of adult male *c739>dPGAM5 IR* flies (*c739-GAL4/UAS-dPGAM5 IR*, *n* = 150; **A**), *OK107>dPGAM5 IR* flies (*UAS-dPGAM5 IR/+; OK107-GAL4/+*, *n* = 90; **B**), *201Y>dPGAM5 IR* flies (*201Y-GAL4/UAS-dPGAM5 IR*, *n* = 70; **C**) and *elav^c155^>dPGAM5 IR* flies (*elav^c155^-GAL4/Y*; *UAS-dPGAM5 IR/+*, *n* = 120; **D**) subjected to HS are shown. *LacZ IR* vs. *dPGAM5 IR* in each group, *p*<0.0001 by the log-rank test.

We then examined whether or not the tissue-specific knockdown of *dPGAM5* could also induce vulnerability of flies to HS. Whereas *dPGAM5* knockdown in various tissues such as the fat body (*yolk-GAL4*), hemocytes (*pxn-GAL4*), eye (*sev-GAL4*), bristle (*sca-GAL4*) and the mediodorsal parts of the thoracic and abdominal segments (*pnr-GAL4*) exerted no effect on the HS response (Fig. S2A–E), *dPGAM5* knockdown in the MB using three different driver strains (*c739-GAL4*, *OK107-GAL4* and *201Y-GAL4*) [12], [13] induced vulnerability in the flies to HS similar to that observed in *PGAM5^1^* flies (Fig. 2A–C).

Consistent with the fact that the MB is an insect brain structure composed primarily of neuronal cell bodies and neurites [14], *dPGAM5* knockdown using the pan-neuronal *elav^c155^-GAL4* driver induced the vulnerability of flies to HS (Fig. 2D). On the other hand, *dPGAM5* knockdown in dopaminergic neurons using the *tyrosine hydroxylase (TH)-GAL4* driver had no effect on the sensitivity of the flies to HS (Fig. S2F), suggesting that the MB, but not necessarily the entire system of neuronal tissues, plays a major role in the HS response in dPGAM5-deficient flies. These findings, taken together, indicate that a deficiency of dPGAM5 in the MB may be responsible for the vulnerability to HS.

### Phosphatase activity of dPGAM5 is crucial for the whole-body HS response

**Figure 3:**
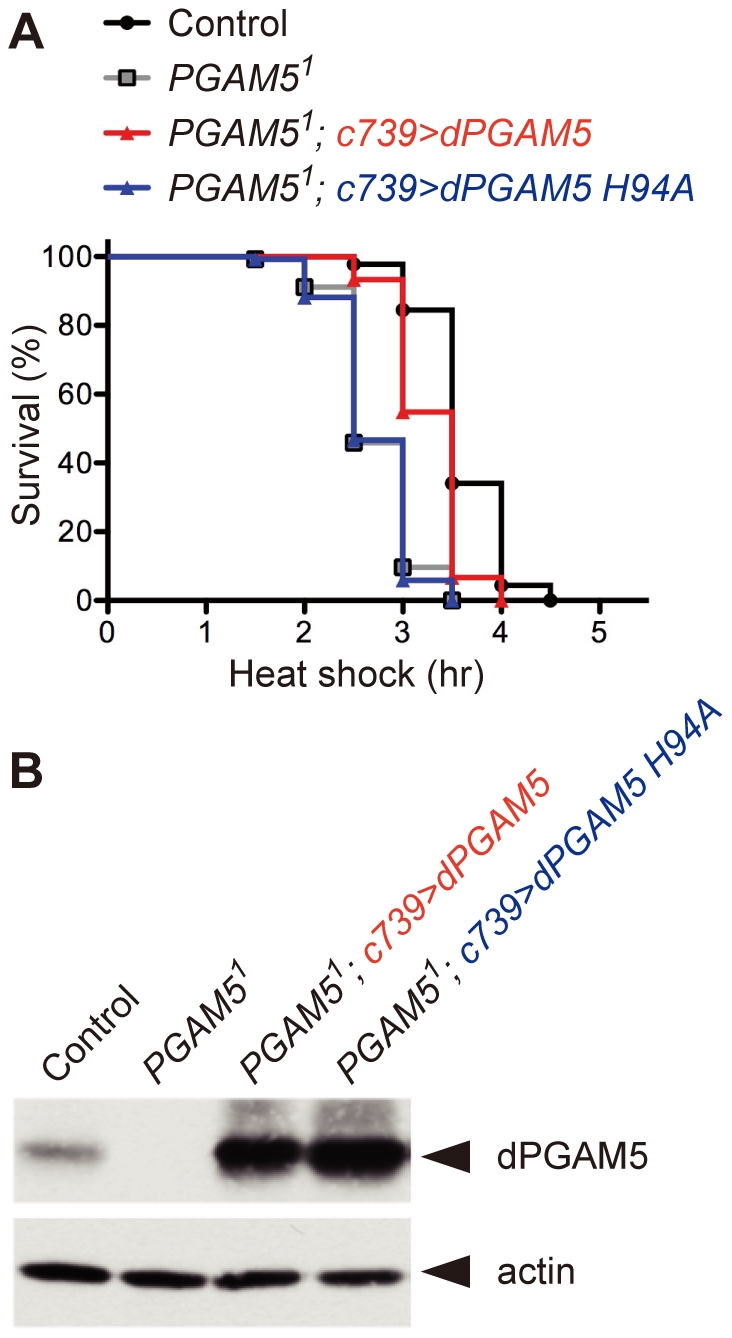
Phosphatase activity of dPGAM5 is crucial for the whole-body HS response. (**A**) Expression of dPGAM5, but not of its phosphatase-inactive mutant, in the MB attenuates the vulnerability of *PGAM5^1^* flies to HS. Survival curves of the indicated adult male flies subjected to HS are shown (*n* = 135). *PGAM5^1^* vs. *PGAM5^1^; c739>dPGAM5*, *p*<0.0001 by the log-rank test. (**B**) *c739-GAL4*-dependent expression of dPGAM5. Protein expression of dPGAM5 in the indicated flies was detected by immunoblotting with dPGAM5 antibody. Actin was also detected as a loading control. The genotypes are *c739-GAL4/+* (Control), *PGAM5^1^/Y; c739-GAL4/+* (*PGAM5^1^*), *PGAM5^1^/Y*; *c739-GAL4/+; UAS-dPGAM5/+* (*PGAM5^1^; c739>dPGAM5*) and *PGAM5^1^/Y; c739- GAL4/+; UAS-dPGAM5 H94A/+* (*PGAM5^1^; c739>dPGAM5 H94A*). All flies were crossed at 22°C and maintained at 25°C after hatching.

To determine whether or not the vulnerability of *PGAM5^1^* flies to HS might indeed be attributable to a loss of *dPGAM5* in the MB, we expressed dPGAM5 selectively in the MB of *PGAM5^1^* flies using the *c739-GAL4* driver (*PGAM5^1^; c739>dPGAM5* flies). The vulnerability of these flies to HS was attenuated compared to that of *PGAM5^1^* flies (Fig. 3A), indicating that the loss of *dPGAM5* in the MB was responsible for the vulnerability of *PGAM5^1^* flies to HS. On the other hand, the phosphatase-inactive mutant of dPGAM5 (H94A) [10], when expressed at a similar level to wild-type dPGAM5 in the MB, had no effect on the sensitivity of *PGAM5^1^* flies to HS (Fig. 3A and B). These results suggest that, depending on its phosphatase activity, dPGAM5 in the MB plays a critical role in the protection of flies from HS.

### dPGAM5 exerts a protective effect against HS by preventing apoptosis in the MB

**Figure 4:**
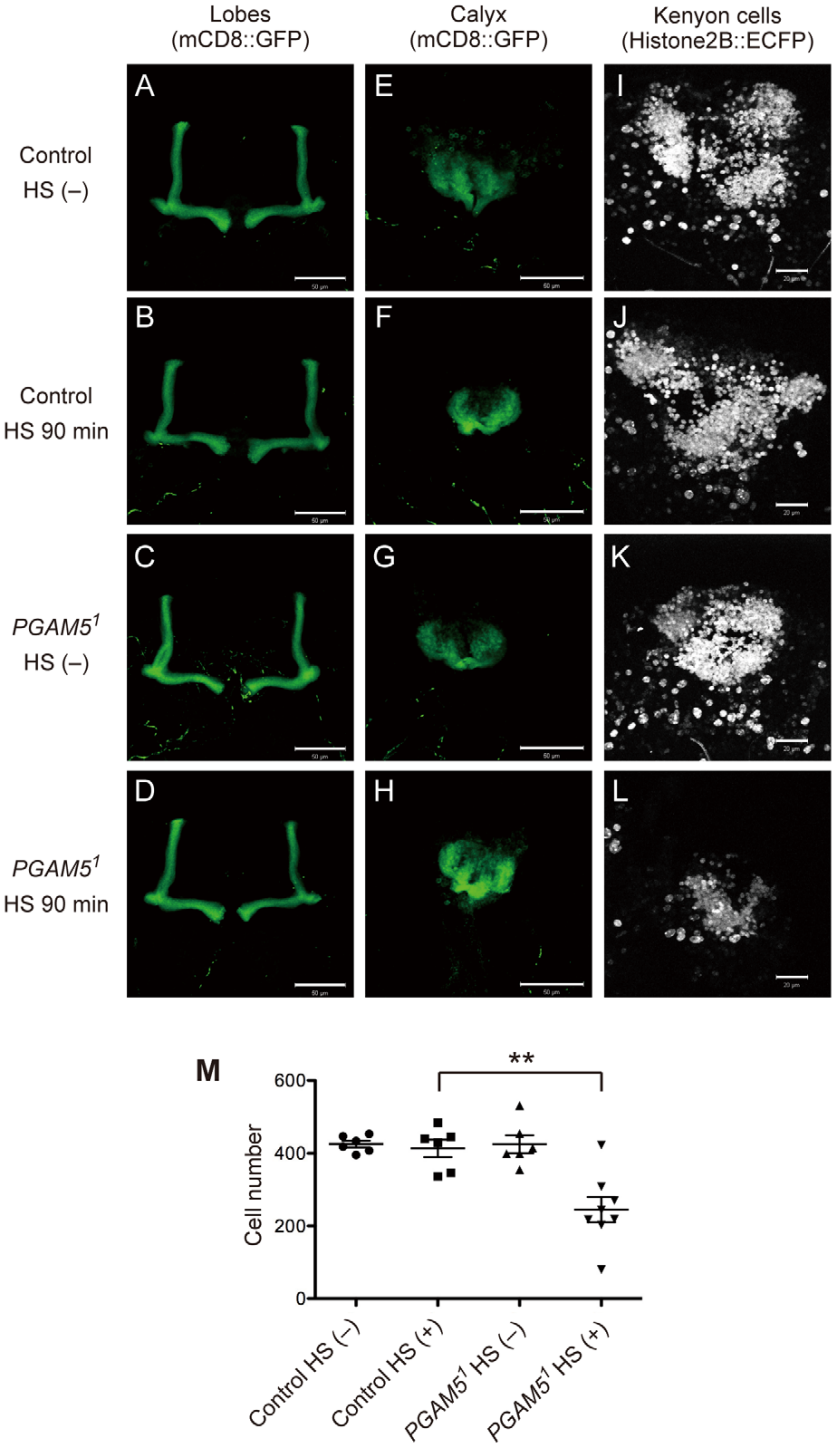
HS induces a rapid degeneration of the nuclei of the MB neurons in the absence of dPGAM5. The number of cell bodies of the KCs decreases in HS-treated *PGAM5^1^* flies. Representative images of lobes (**A**–**D**) and calyxes (**E**–**H**) visualized by mCD8::GFP are shown. The genotypes are *c739-GAL4/+; UAS-mCD8::GFP/+* (Control) and *PGAM5^1^*/*Y; c739*-*Gal4*/+; *UAS*-*mCD8::GFP*/+ (*PGAM5^1^*). Scale bar (**A**–**H**) = 50 µm. Representative images of the KC nuclei visualized by Histone2B::ECFP (**I**–**L**; scale bar = 20 µm) and the total number of KC nuclei (**M**) are shown. The latter was calculated by summing up the number counted manually in every tenth section, and the results were shown as the mean cell number per fly ± s.e.m. [*n* = 6, Control HS (−) and (+) and *PGAM5^1^* HS (−); *n* = 8, *PGAM5^1^* HS (+)]. ** P<0.01, unpaired t-test (**M**). The genotypes are *c739-GAL4*/*UAS-Histone2B::ECFP* (Control) and *PGAM5^1^*/*Y*; *c739-GAL4*/*UAS-Histone2B::ECFP* (*PGAM5^1^*).

**Figure 5:**
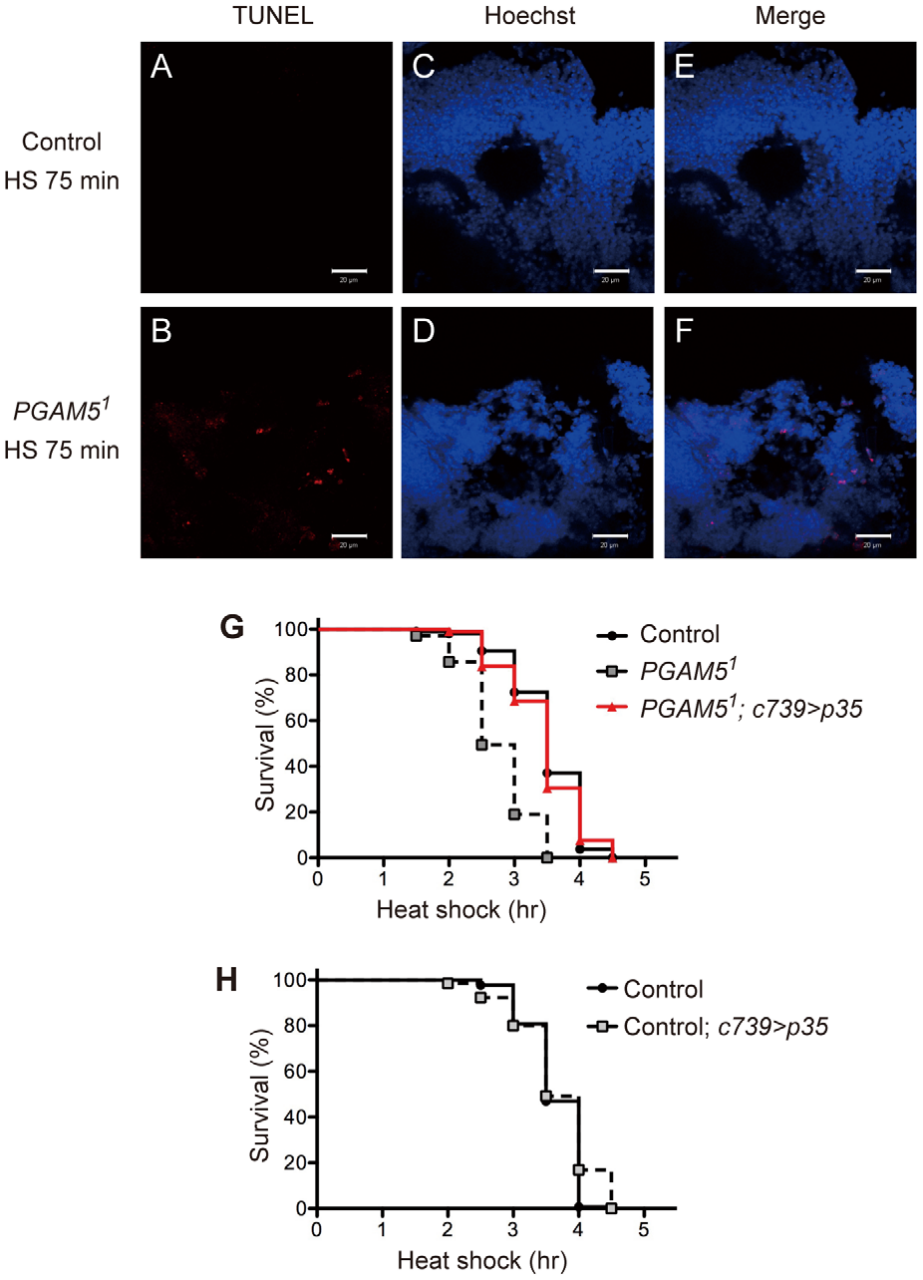
dPGAM5 exerts its protective effect against HS by preventing apoptosis in the MB. (**A–F**) TUNEL-positive KCs are detected in the MB of HS-treated *PGAM5^1^* flies, but not in that of control flies. TUNEL staining of the MB of control flies (**A**) and *PGAM5^1^* flies (**B**) treated with HS for 75 min is shown. Counter nuclear staining with Hoechst33258 (**C** and **D**) and merged images of TUNEL and Hoechst 33258 staining (**E** and **F**) are also shown. Scale bar (**A**–**F**) = 20 µm. The genotypes are *c739-GAL4/UAS-Histone2B::ECFP* (Control) and *PGAM5^1^/Y; c739-GAL4/UAS-Histone2B::ECFP* (*PGAM5^1^*). (**G**) Expression of p35 in the MB attenuates the vulnerability of *PGAM5^1^* flies to HS. Survival curves of the indicated adult male flies subjected to HS are shown (*n* = 105). *PGAM5^1^* vs. *PGAM5^1^; c739>p35*, *p*<0.0001 by the log-rank test. The genotypes are *c739-GAL4/+* (Control), *PGAM5^1^/Y; c739-GAL4/+* (*PGAM5^1^*) and *PGAM5^1^/Y; c739-GAL4/+; UAS-p35/+* (*PGAM5^1^; c739>p35*). (**H**) Expression of p35 in the MB does not affect the response of control flies to HS. Survival curves of the indicated adult male flies subjected to HS are shown (*n* = 130). The genotypes are *c739-GAL4/+* (Control) and *c739-GAL4/+; UAS-p35/+* (Control; *c739>p35*).

To elucidate the role of dPGAM5 in the MB, we next examined HS-induced histological changes in the MB. The MB consists primarily of a cluster of intrinsic neurons referred to as Kenyon cells (KCs), together with their dendritic branches (calyx) and axonal bundle (pedunculus). At the end of the pedunculus, the axons bifurcate dorsally and medially to form the vertical and medial lobes, respectively [14]. When the lobes and calyxes were observed by MB-specific expression of a fusion protein between a transmembrane protein CD8 and GFP (mCD8-GFP), which labels the cell surface, HS-induced morphological changes of these regions were induced in neither control flies nor *PGAM5^1^* flies (Fig. 4A–H). However, when the nuclei of a subset of KCs were visualized by *c739-GAL4*-dependend expression of the nucleus-targeting Histone2B::ECFP, which was previously shown to detect nuclear condensation and morphological changes that accompany cell death [15], the number of KC nuclei decreased in *PGAM5^1^* flies, but not in control flies, upon 90-min HS treatment (Fig. 4I–M). Consistent with this, Hoechst 33258 staining of the nuclei of KCs in *PGAM5^1^* flies was reduced compared to that in control flies already after 75-min HS treatment, which is when *PGAM5^1^* flies started to die (Fig. 5C and D). These results suggested that HS induced a rapid degeneration of the nuclei of the MB neurons in the absence of dPGAM5, although the overall structures of the neurons were relatively preserved at the same time point.

Based on the previous finding that HS induced apoptosis in the wing imaginal disc of third-instar larvae [6], we examined whether or not apoptosis is involved in the HS-induced degeneration of the MB neurons of adult flies. When HS-induced apoptosis in the MB was examined by TUNEL staining, TUNEL-positive KCs were observed in *PGAM5^1^* flies, but not in control flies, after 75-min HS treatment (Fig. 5A and B). At the same time point, TUNEL-positive cells in other brain regions we examined, such as the optic lobe, were detected in neither control flies nor *PGAM5^1^* flies (Fig. S3), suggesting that TUNEL-positive cells were almost restricted to the MB in HS-treated *PGAM5^1^* flies. Even after 150-min HS treatment, i.e., when approximately 20% of the control flies had died, only a negligible amount of TUNEL-positive cells was detected in the MB of control flies (data not shown). These results indicated that apoptosis was induced in the MB of *PGAM5^1^* flies to a much greater extent than in that of control flies. Nevertheless, the number of TUNEL-positive cells in the MB of *PGAM5^1^* flies was much smaller than we expected from the obvious reduction in the number of Histone2B::ECFP-labeled nuclei after 90-min HS treatment (Fig. 4); this may be because each apoptotic cell only transiently reacted to TUNEL according to a rapid degeneration of the nuclei in the absence of dPGAM5. Taken together, apoptosis in the MB may be involved in HS-induced death of individual *PGAM5^1^* flies but not of control flies.

We next examined whether or not apoptosis in the MB is indeed responsible for the vulnerability of *PGAM5^1^* flies to HS. To this end, we suppressed HS-induced apoptosis in the MB by selective expression of the caspase inhibitor protein p35 in the MB of *PGAM5^1^* flies. We found that expression of p35 in the MB of *PGAM5^1^* flies decreased their vulnerability, and the resulting flies exhibited a similar response to HS to that of control flies (Fig. 5G). On the other hand, expression of p35 in the MB of control flies did not affect their response to HS (Fig. 5H), further supporting that apoptosis in the MB was not involved in HS-induced death of individual control flies. Taken together, the findings indicate that dPGAM5 does appear to exert a protective effect against HS by preventing apoptosis in the MB.

### The MB gains a toxic function, rather than loses a protective function, in response to HS in the absence of dPGAM5

**Figure 6:**
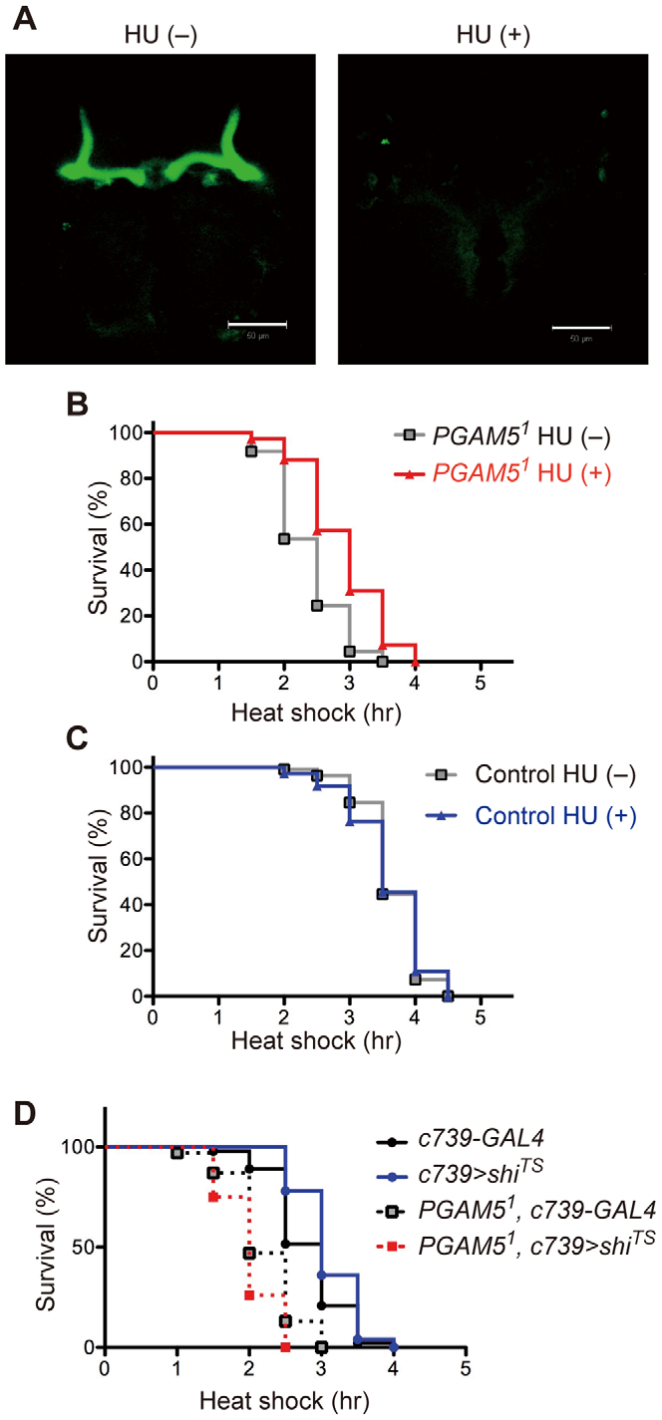
The MB gains a toxic function, rather than loses a protective function, in response to HS in the absence of dPGAM5. (**A**) Ablation of the MB. Ablation of the MB in adult flies pretreated with hydroxyurea (HU) at the larval stage was confirmed by MB-specific expression of mCD8::GFP. Scale bar = 50 µm. The genotype is *c739-GAL4/+; UAS-mCD8::GFP/+*. (**B**) Ablation of the MB attenuates the vulnerability of *PGAM5^1^* flies to HS. Survival curves of adult male *PGAM5^1^* flies (*y^1^*, *PGAM5^1^/Y*) untreated (−) or treated (+) with HU are shown (*n* = 110). *p*<0.0001 by the log-rank test. (**C**) Ablation of the MB does not affect the response of control flies to HS. Survival curves of adult male control (*y^1^w^1^/Y*) flies untreated (−) or treated (+) with HU are shown (*n* = 110). (**D**) Suppression of neurotransmission does not decrease vulnerability of *PGAM5^1^* flies to HS. Survival curves of the indicated adult male flies (day 9–15) subjected to HS are shown. *c739-GAL4* flies (*+/Y; c739-GAL4/+; Sb/+*, *n* = 91), *c739>shi^TS^* flies (*+/Y; c739-GAL4/+; UAS- shi^TS^/+*, *n* = 100), *PGAM5^1^, c739-GAL4* flies (*PGAM5^1^/Y; c739-GAL4/+; Sb/+*, *n* = 100) and *PGAM5^1^, c739>shi^TS^* flies (*PGAM5^1^/Y; c739-GAL4/+; UAS-shi^ TS^/+*, *n* = 100) were examined.

As regards the role of the MB in the whole-body HS response, two possibilities were considered: 1) the MB exerts an apoptosis-sensitive protective function against HS, and 2) the MB acquires an apoptosis-dependent toxic function. To address these possibilities, we chemically ablated the MB by treating flies with hydroxyurea (HU) at the larval stage. Flies lacking the MB are known to develop without any obvious abnormalities [16]–[18]; here, we confirmed that the MB was indeed ablated in adult flies pretreated with HU (Fig. 6A). Interestingly, *PGAM5^1^* flies lacking the MB were more resistant to HS than those possessing the MB (Fig. 6B), whereas control flies lacking the MB exhibited a similar response to HS to that of flies retaining the MB (Fig. 6C). These results once again demonstrated the critical role played by the MB in the whole-body HS response and suggest that the MB gains a toxic function in the absence of dPGAM5, instead of losing a protective function in response to HS.

Taking account of the function of the MB as a neuronal tissue, we raised the possibility that the toxic function of the MB in HS-treated *PGAM5^1^* flies might depend on synaptic signaling from KCs to other neuronal cells. To address this possibility, we used a temperature sensitive allele of the *Drosophila* dynamin gene *shibire* (*shi^TS^*), which loses its activity at 30°C and above and suppresses synaptic neurotransmission [19]–[21]. However, suppression of neurotransmission during the HS treatment by expression of *shi^TS^* in the MB of *PGAM5^1^* flies did not decrease but rather increased their vulnerability to HS (Fig. 6D), suggesting that the toxic function of the MB does not depend on synaptic signaling. Thus, the toxic function gained by the MB in response to HS in the absence of dPGAM5 may depend, not on synaptic transmission, but rather on the passive release of toxic factor(s) from apoptotic neurons.

### Locomotor activity is reduced in dPGAM5-deficient flies

**Figure 7:**
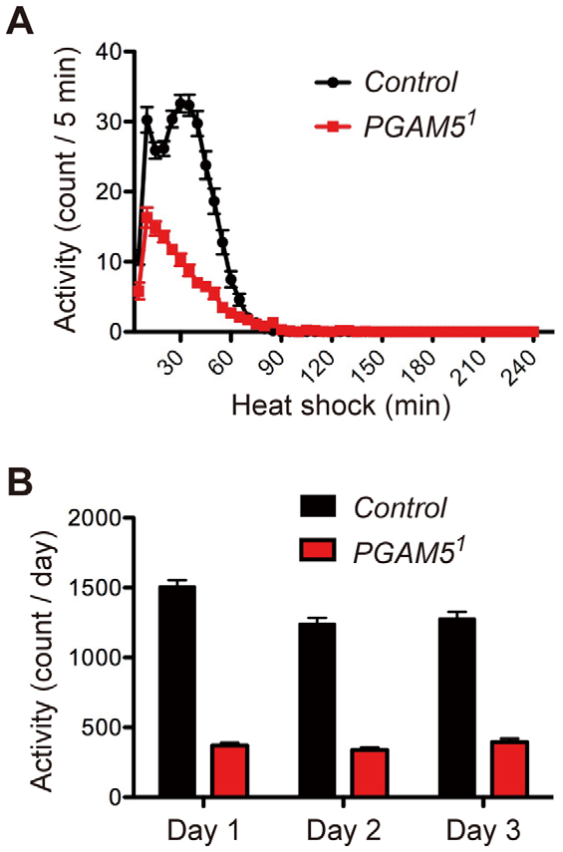
Locomotor activity is reduced in dPGAM5-deficient flies. *PGAM5^1^* flies are less active than control flies under both HS (37°C; **A**) and unstressed (25°C; **B**) conditions. Locomotor activity was monitored using the *Drosophila* activity monitor (DAM) system as described in the Materials and Methods. Data are shown as the mean counts ± s.e.m of total flies examined for each genotype [Control, *n* = 77 and 79; *PGAM5^1^*, n = 76 and 78; at 37°C and 25°C, respectively]. The genotypes are *y^1^w^1^/Y* (Control) and *PGAM5^1^/Y* (*PGAM5^1^*).

Since the MB have been shown to influence locomotor activity, as well as higher brain functions such as olfactory learning and memory [17], [18], we examined locomotor activity of *PGAM5^1^* flies during the HS treatment using the *Drosophila* activity monitor (DAM) system (Fig. 7A). We found that control flies exhibited increased locomotor activity immediately after they were subjected to HS, which peaked around 30 min and then decreased within 60 min. On the other hand, *PGAM5^1^* flies were overall less active than control flies, and their activity peaked immediately after they were subjected to HS and gradually decreased thereafter within 60 min. We next examined whether or not this reduced locomotor activity of *PGAM5^1^* flies was restricted to their HS response (Fig. 7B). To this end, we monitored locomotor activity of control and *PGAM5^1^* flies under normal unstressed conditions for three days. We found that activity of *PGAM5^1^* flies was constantly lower than that of control flies, suggesting that locomotor activity of *PGAM5^1^* flies was reduced under both unstressed and stressed conditions. Although it is difficult from these data to determine if the reduced HS-induced locomotor activity affects the increased vulnerability of *PGAM5^1^* flies to HS, dPGAM5, possibly in the MB, may influence locomotor activity of flies even under unstressed conditions.

## Discussion

The analyses of dPGAM5-deficient flies in this study revealed the unexpected finding that the MB, a restricted brain structure, plays a critical role in the whole-body response of flies to HS. Consistent with the proposed central roles of the MB in higher brain functions such as olfactory learning and memory [22], the MB appears to be dispensable for the HS response observed in control flies, in that the elimination of the MB from control flies did not affect their response to HS. However, once protection by putative guardians such as dPGAM5 is lost or attenuated, the cells of the MB undergo apoptosis, rendering flies vulnerable to HS, most likely due to some toxic effect(s) rather than to the loss of a protective effect. Our findings suggest that the strict protection of the MB is a prerequisite for proper resistance to HS, at least in adult flies. As regards the responses of larvae and pupae, it has previously been shown that HS during development selectively disrupts MB anatomy, resulting in an impairment in the learning of odors [23], although it should be noted that the involvement of dPGAM5 in HS during fly development has not yet been investigated.

The dependence of the protective function of dPGAM5 against HS on phosphatase activity is an initial and important insight into the actual function of PGAM5 phosphatase activity *in vivo*. However, the protective effect of dPGAM5 found here was rather unexpected, as we had recently noted that dPGAM5 acts as an exacerbating factor in the *Drosophila* model of Parkinson's disease induced by PINK1 mutation [11]. In those model flies, tissues with relatively high-energy demands, such as flight muscles and dopaminergic neurons, are known to be preferentially affected. One possible explanation for these opposing roles of dPGAM5 could be that in such fly tissues, dPGAM5 might exert deleterious effects in the context of *PINK1* loss, whereas in other tissues, including the MB, dPGAM5 might generally exert protective effects against various stressors. In fact, dPGAM5 in the MB appears to exert protective effects against HS independently of dPINK1, although dPINK1 also protected flies from HS.

Although at present we do not have a full grasp of the role of dPGAM5 in the HS response at the molecular level, it is possible that dPGAM5 may “sense” HS-induced mitochondrial aberrations such as the accumulation of heat-damaged proteins within the mitochondria, and it may transduce signals from the damaged mitochondria to other cellular compartments. Further elucidation of the molecular functions of dPGAM5 and the potential toxic roles of the MB in various states of deterioration will shed light on the regulation of the HS response at the level of the organism in flies, and hopefully in higher organisms as well.

## Materials and Methods

### Flies

Fly cultures and crosses were carried out on normal growth medium (10% glucose, 4% dry yeast, 4% cornmeal and 0.9% agar) at 25°C, unless otherwise stated. Transgenic flies harboring *UAS-dPGAM5* and *UAS-dPGAM5 H94A* were generated by standard P element-mediated transformation (BestGene Inc.). The following fly strains were used in this study: *PINK1^B9^*, revertant *PINK1^REV^* [24]; *PGAM5^1^, PINK1^B9^* [11]; *UAS-LacZ IR* [25]; *pnr-GAL4* [26]; *pxn-GAL4* [27]; *sca-GAL4* [28]; *yolk-GAL4* [29]; *TH-GAL4* [30]; UAS-*shi^TS^* [19]; *sev-GAL4* [31], *repo-GAL4* [32], *OK107-GAL4* [13], *201Y-GAL4*, *c739-GAL4* [12], *UAS-mCD8::GFP* [33], *UAS-Histone2B::ECFP* [15], *da-GAL4*, *hs-GAL4* and *elav^c155^-GAL4* (Bloomington *Drosophila* Stock Center); and *UAS-dPGAM5 IR* (R-6) (NIG-FLY stock center).

### Stress treatments

Adult flies (aged 3–5 days) were subjected to various stressors, and live flies were counted on the basis of movement following manipulation of vials as previously described [34]. For the oxidative stress condition, flies were placed in vials containing a medium containing 0.1% H_2_O_2_, 1% sucrose and 1.3% agar at 25°C, and live flies were counted daily. For the starvation condition, flies were placed in empty vials at 25°C, and live flies were counted every 3 h. For HS, flies in vials containing normal growth medium were placed in a 37°C water bath, and live flies were counted every 30 min. For the assay of *hs>LacZ-IR* and *hs>PGAM5-IR* flies, IR RNA was induced in flies (aged 12–17 days) two days prior to sustained HS by two cycles of HS pretreatment, each of which was composed of 37°C for 30 min, 25°C for 5 hr, 37°C for 30 min and 25°C for 18 hr.

### Immunoblotting

Flies were lysed with RIPA buffer (50 mM Tris-HCl pH 8.0, 150 mM NaCl, 1% NP40, 0.5% deoxycholate and 0.1% SDS), and lysates were resolved on SDS-PAGE and electroblotted onto PVDF membranes. After blocking with 5% skim milk in TBS-T (50 mM Tris-HCl, pH 8.0, 150 mM NaCl and 0.05% Tween 20), the membranes were probed with dPGAM5 polyclonal antibody [11] and actin monoclonal antibody (Sigma). The antibody-antigen complexes were detected using the ECL system (GE Healthcare).

### Ablation of the MB

Immediately after hatching, larvae were fed a heat-killed yeast suspension containing 25 mM hydroxyurea (HU; Invitrogen) for 4 h. Larvae were then washed in distilled water and cultured on normal growth medium at 25°C [16].

### Histology

Brains dissected from adult flies were fixed with 4% paraformaldehyde in 0.3% Triton X-100/PBS (PBT) for 20 min at room temperature (RT). After three washes with a buffer containing 54 mM NaCl, 40 mM KCl, 7.4 mM MgSO_4_, 2.4 mM CaCl_2_, 4.8 mM tricine, 0.36% (w/v) glucose, 1.7% (w/v) sucrose and 0.3% Triton X-100 (20 min each), the brains were incubated with 5 µg/ml proteinase K (Nacalai Tesque) for 5 min, followed by three washes with PBT (20 min each) at RT. TUNEL staining was performed using the *In Situ* Cell Death Detection Kit TMR-Red (Roche) for 1 h at 37°C. Brains were then counterstained with 400 ng/ml Hoechst 33258 for 3 h at RT. Fluorescence images were acquired with an LSM510 confocal microscope (Carl Zeiss). For counting the KCs, Z-series confocal images were collected to cover the KC perikarya cluster (0.75 µm virtual sections), and the total number of KC nuclei visualized by Histone2B::ECFP was calculated by summing up the number counted manually in every tenth section.

### Locomotor activity assay

Male flies of each genotype (day 5–10) were entrained to a 12 hr light: 12 hr dark cycle (LD12:12) at 25°C for at least 2 days. Flies were transferred individually to glass tubes containing food at one end. Locomotor activity was monitored by recording infrared beam crossings by individual flies using the *Drosophila* activity monitoring (DAM) system (Trikinetics) [35], [36]. For HS conditions, flies in glass tubes were placed in an air incubator at 37°C, and their locomotor activity was monitored every 5 min. For unstressed conditions, data were collected continuously for at least 3 days under LD12:12 conditions at 25°C.

## Supporting Information

Figure S1 **Lifespan assay of** ***PGAM5^1^*** **flies.** For each group, 200 adult male control (*y^1^w^1^/Y*) and *PGAM5^1^* (*y^1^*, *PGAM5^1^/Y*) flies were maintained on normal growth medium (10% glucose, 4% dry yeast, 4% cornmeal and 0.9% agar) at 25°C, and live flies were counted daily on the basis of movement following manipulation of the vials. Flies were transferred to vials containing fresh medium every three or four days.
(TIF)

Figure S2 **Effects of tissue-specific knockdown of** ***dPGAM5*** **on the response of flies to HS.** Survival curves of the indicated adult male flies subjected to HS are shown. (**A**) *yolk-GAL4/UAS-LacZ IR* (*yolk>LacZ IR*), *yolk-GAL4/UAS-dPGAM5 IR* (*yolk>dPGAM5 IR*), n = 90. (**B**) *pxn-GAL4/UAS-LacZ IR* (*pxn>LacZ IR*), *pxn-GAL4/UAS-dPGAM5 IR* (*pxn>dPGAM5 IR*), n = 105. (**C**) *sev-GAL4/UAS-LacZ IR* (*sev>LacZ IR*), *sev-GAL4/UAS-dPGAM5 IR* (*sev>dPGAM5 IR*), n = 100. (**D**) *sca-GAL4/UAS-LacZ IR* (sca>*LacZ IR*), *sca-GAL4/UAS-dPGAM5 IR* (*sca>dPGAM5 IR*), n = 120. (**E**) *UAS-LacZ IR/+; pnr-GAL4/+* (*pnr>LacZ IR*), *UAS-dPGAM5 IR/+; pnr-GAL4/+* (*pnr>dPGAM5 IR*), n = 75. (**F**) *UAS-LacZ IR/+; TH-GAL4/+* (*TH>LacZ IR*), *UAS-dPGAM5 IR/+; TH-GAL4/+* (*TH>dPGAM5 IR*), n = 60.
(TIF)

Figure S3 **HS-induced TUNEL-positive cells in the optic lobe are detected in neither control flies nor** ***PGAM5^1^*** **flies.** Hoechst33258 staining (left panels) and TUNEL staining (right panels) of the optic lobe of control flies (upper panels) and *PGAM5^1^* flies (lower panels) treated with HS for 75 min are shown. Scale bar = 20 µm. The genotypes are *c739-GAL4/UAS-Histone2B::ECFP* (Control) and *PGAM5^1^/Y; c739-GAL4/UAS-Histone2B::ECFP* (*PGAM5^1^*).
(TIF)
