# Supplementary figures and images for: Prevention of Apoptosis by Mitochondrial Phosphatase PGAM5 in the Mushroom Body Is Crucial for Heat Shock Resistance in Drosophila melanogaster
Source: PLoS One. 2012 Feb 7;7(2):e30265. doi: 10.1371/journal.pone.0030265 (PMC3274544; doi:10.1371/journal.pone.0030265)

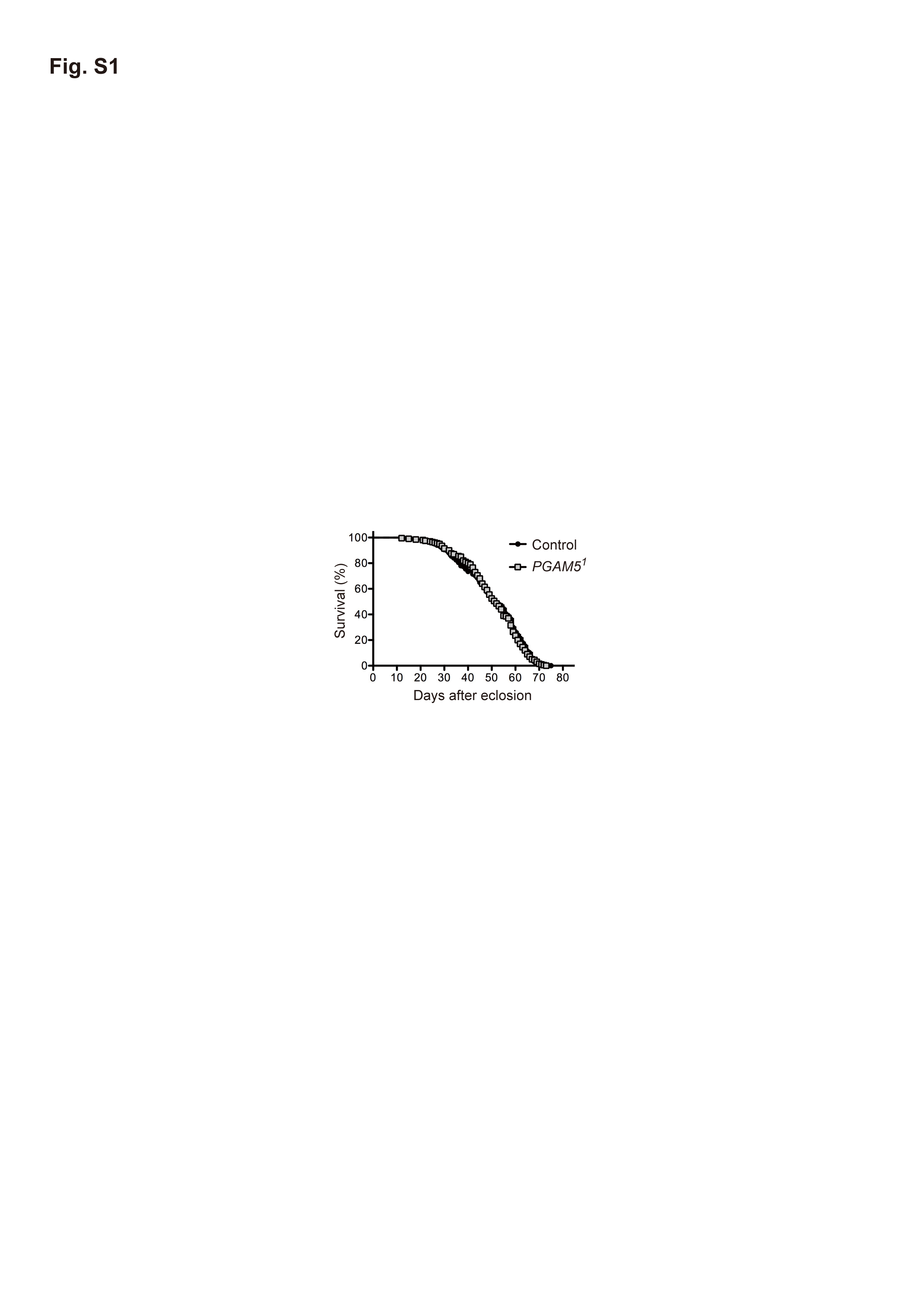

Supplement: Figure S1 — Lifespan assay of PGAM51 flies. For each group, 200 adult male control (y1w1/Y) and PGAM51 (y1, PGAM51/Y) flies were maintained on normal growth medium (10% glucose, 4% dry yeast, 4% cornmeal and 0.9% agar) at 25°C, and live flies were counted daily on the basis of movement following manipulation of the vials. Flies were transferred to vials containing fresh medium every three or four days. (TIF) [file pone.0030265.s001.tif]

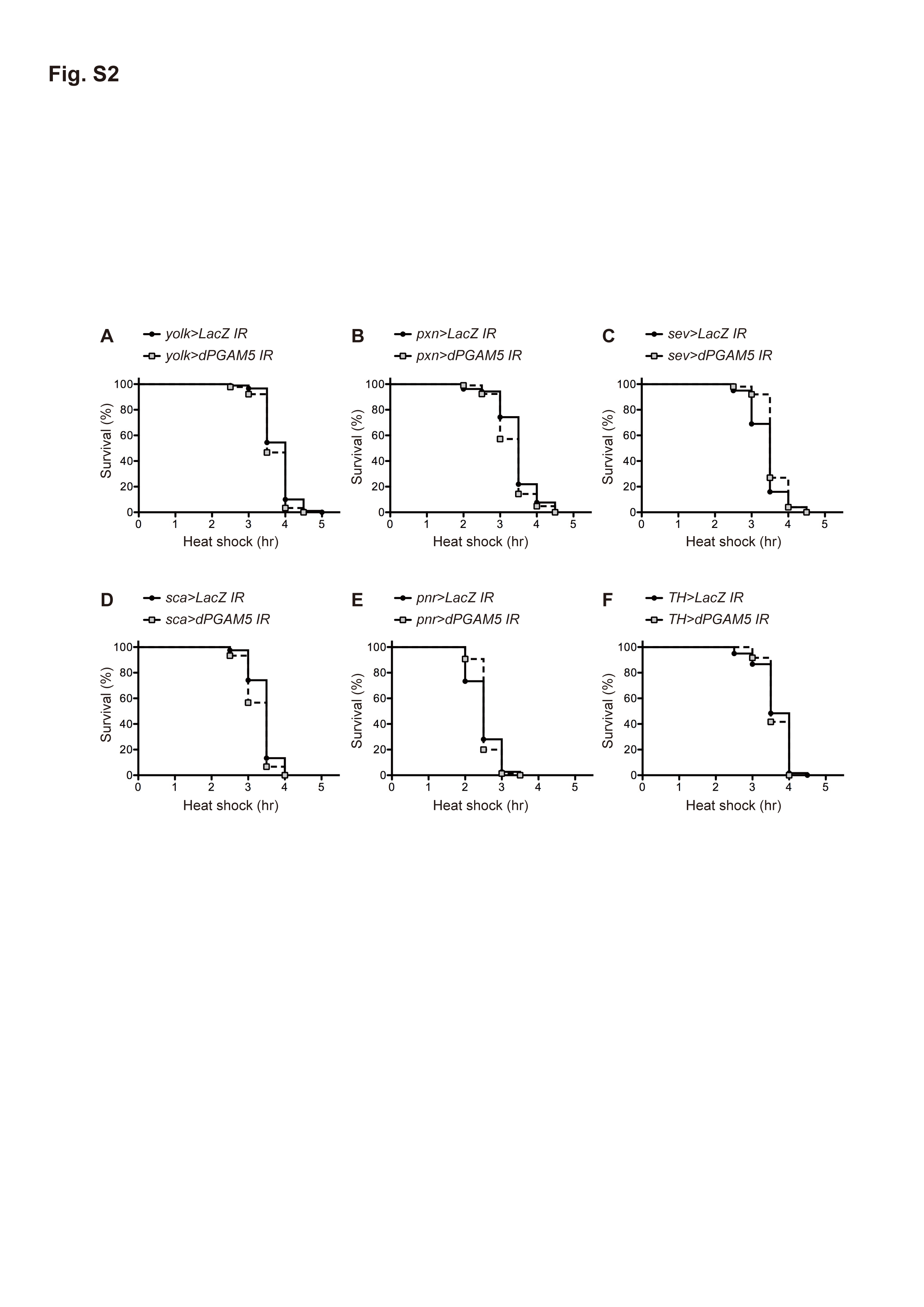

Supplement: Figure S2 — Effects of tissue-specific knockdown of dPGAM5 on the response of flies to HS. Survival curves of the indicated adult male flies subjected to HS are shown. (A) yolk-GAL4/UAS-LacZ IR (yolk>LacZ IR), yolk-GAL4/UAS-dPGAM5 IR (yolk>dPGAM5 IR), n = 90. (B) pxn-GAL4/UAS-LacZ IR (pxn>LacZ IR), pxn-GAL4/UAS-dPGAM5 IR (pxn>dPGAM5 IR), n = 105. (C) sev-GAL4/UAS-LacZ IR (sev>LacZ IR), sev-GAL4/UAS-dPGAM5 IR (sev>dPGAM5 IR), n = 100. (D) sca-GAL4/UAS-LacZ IR (sca>LacZ IR), sca-GAL4/UAS-dPGAM5 IR (sca>dPGAM5 IR), n = 120. (E) UAS-LacZ IR/+; pnr-GAL4/+ (pnr>LacZ IR), UAS-dPGAM5 IR/+; pnr-GAL4/+ (pnr>dPGAM5 IR), n = 75. (F) UAS-LacZ IR/+; TH-GAL4/+ (TH>LacZ IR), UAS-dPGAM5 IR/+; TH-GAL4/+ (TH>dPGAM5 IR), n = 60. (TIF) [file pone.0030265.s002.tif]

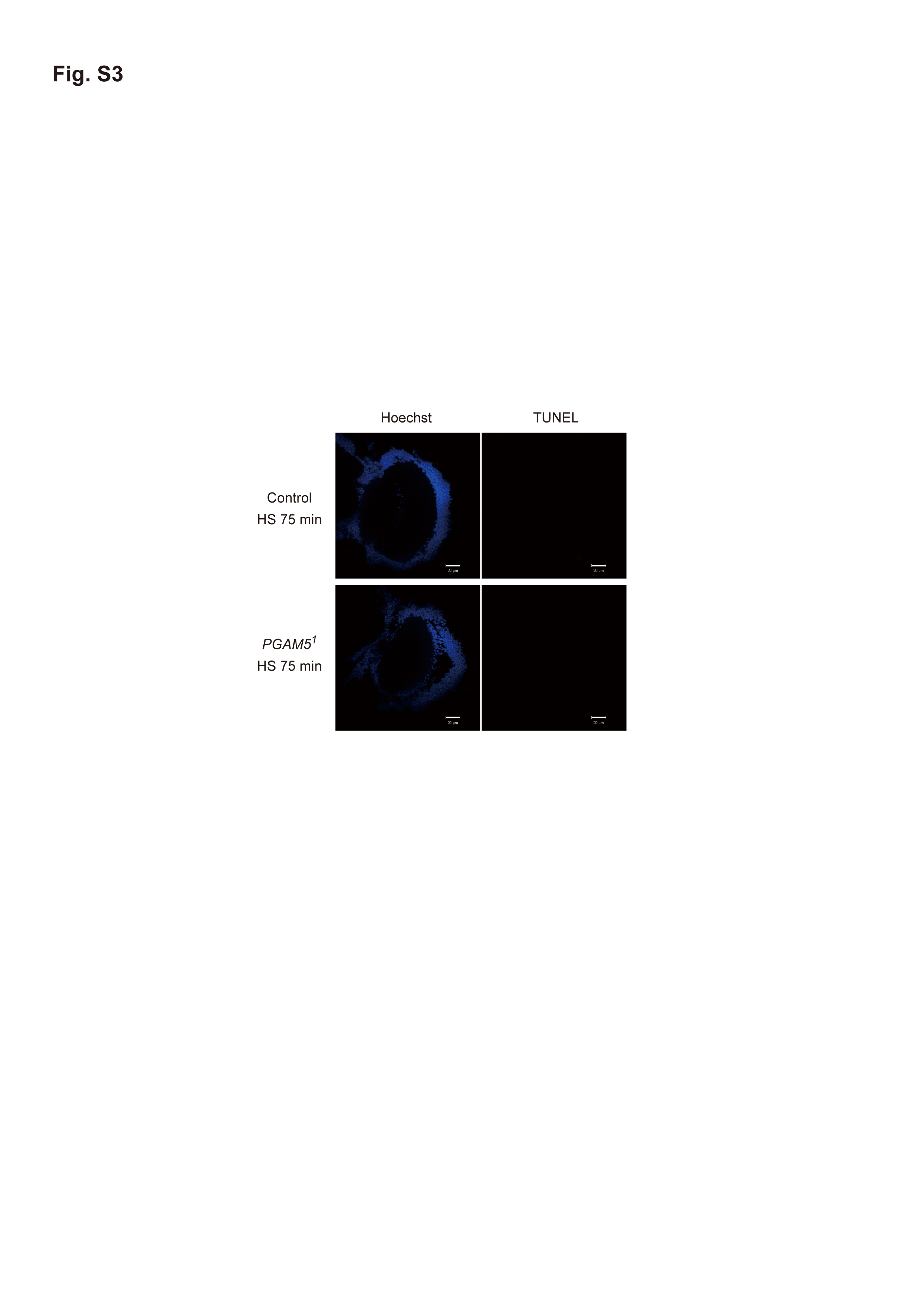

Supplement: Figure S3 — HS-induced TUNEL-positive cells in the optic lobe are detected in neither control flies nor PGAM51 flies. Hoechst33258 staining (left panels) and TUNEL staining (right panels) of the optic lobe of control flies (upper panels) and PGAM51 flies (lower panels) treated with HS for 75 min are shown. Scale bar = 20 µm. The genotypes are c739-GAL4/UAS-Histone2B::ECFP (Control) and PGAM51/Y; c739-GAL4/UAS-Histone2B::ECFP (PGAM51). (TIF) [file pone.0030265.s003.tif]
